# Intracranial bleeding in patients with traumatic brain injury: A prognostic study

**DOI:** 10.1186/1471-227X-9-15

**Published:** 2009-08-03

**Authors:** Pablo Perel, Ian Roberts, Omar Bouamra, Maralyn Woodford, Jane Mooney, Fiona Lecky

**Affiliations:** 1 Epidemiology and Population Health Department, London School of Hygiene & Tropical Medicine, London, UK; 2 Trauma Audit and Research Network, Occupational and Environmental Health Research Group, School of Translational Medicine, University Of Manchester, Manchester, UK

## Abstract

**Background:**

Intracranial bleeding (IB) is a common and serious consequence of traumatic brain injury (TBI). IB can be classified according to the location into: epidural haemorrhage (EDH) subdural haemorrhage (SDH) intraparenchymal haemorrhage (IPH) and subarachnoid haemorrhage (SAH). Studies involving repeated CT scanning of TBI patients have found that IB can develop or expand in the 48 hours after injury. If IB enlarges after hospital admission and larger bleeds have a worse prognosis, this would provide a therapeutic rationale for treatments to prevent increase in the extent of bleeding. We analysed data from the Trauma Audit & Research Network (TARN), a large European trauma registry, to evaluate the association between the size of IB and mortality in patients with TBI.

**Methods:**

We analysed 13,962 patients presenting to TARN participating hospitals between 2001 and 2008 with a Glasgow Coma Score (GCS) less than 15 at presentation or any head injury with Abbreviated Injury Scale (AIS) severity code 3 and above. The extent of intracranial bleeding was determined by the AIS code. Potential confounders were age, presenting Glasgow Coma Score, mechanism of injury, presence and nature of other brain injuries, and presence of extra-cranial injuries. The outcomes were in-hospital mortality and haematoma evacuation. We conducted a multivariable logistic regression analysis to evaluate the independent effect of large and small size of IB, in comparison with no bleeding, on patient outcomes. We also conducted a multivariable logistic regression analysis to assess the independent effect on mortality of large IB in comparison with small IB.

**Results:**

Almost 46% of patients had at some type of IB. Subdural haemorrhages were present in 30% of the patients, with epidural and intraparenchymal present in approximately 22% each. After adjusting for potential confounders, we found that large IB, wherever located, was associated with increased mortality in comparison with no bleeding. We also found that large IB was associated with an increased risk of mortality in comparison with small IB. The odds ratio for mortality for large SDH, IPH and EDH, in comparison with small bleeds, were: 3.41 (95% CI: 2.68-4.33), 3.47 (95% CI: 2.26-5.33) and 2.86 (95% CI: 1.86-4.38) respectively.

**Conclusion:**

Large EDH, SDH and IPH are associated with a substantially higher probability of hospital mortality in comparison with small IB. However, the limitations of our data, such as the large proportion of missing data and lack of data on other confounding factors, such as localization of the bleeding, make the results of this report only explanatory. Future studies should also evaluate the effect of IB size on functional outcomes.

## Background

Intracranial bleeding (IB) is a common and serious consequence of traumatic brain injury (TBI). In the MRC CRASH trial, which included mild, moderate and severe TBI patients, 56% of trial participants had at least one IB. [1] The frequency of IB varies with TBI severity, age, presence or absence of compound skull fracture, and the anatomical site of injury (frontal, temporo-parietal, occipital). [2] IB can be classified according to the location into epidural haemorrhage (EDH) subdural haemorrhage (SDH) intraparenchymal haemorrhage (IPH) and subarachnoid haemorrhage (SAH).

A review by the Brain Trauma Foundation found that all types of IB are associated with a worse prognosis, with increased in-hospital mortality and disability at six months. [3] Analysis of data from the CRASH trial showed that subarachnoid bleeding and non evacuated haematoma were independently associated with a worse outcome at 2 weeks and 6 months. [4] Similarly, the IMPACT study found that after controlling for age, Glasgow Coma Score (GCS) motor score and pupil reactions, subarachnoid and subdural bleeding doubled the odds of poor outcome at six months. [5]

Some studies involving repeated CT scanning of patients with TBI have found that intracranial bleeding can develop or expand in the 24-48 hours after injury. These findings have generated interest in potential therapeutic approaches, such as haemostatic drugs, that could prevent or decrease the growth of IB. [6]

If IB enlarges after hospital admission and larger bleeds have a worse prognosis, this would strengthen the therapeutic rationale for agents to prevent an increase in the extent of bleeding. Although there have been some studies on the association between size of IB and prognosis, the empirical evidence is limited, most studies having small sample sizes and restricted populations. [7-10]

We analysed data from the Trauma Audit & Research Network (TARN), a large European trauma registry, to evaluate the association between the size of IB and, mortality and haematoma evacuation, in patients with TBI.

## Methods

### Sample

TARN was established in 1989 to benchmark and improve hospital trauma care (using case fatality measures). Membership is voluntary and includes 60% of hospitals receiving trauma patients in England and Wales and some hospitals in European centres. TARN has ethical approval for research on anonymized data through the patient information access group (PIAG3-04(E) 2006). The TARN database contains no patient identifiers

Data are collected on patients who arrive at hospital alive and meet any of the subsequent criteria:

- Death from injury at any point during admission

- stay in hospital for longer than 3 days

- require intensive or high dependency care

- require inter - hospital transfer for specialist care

Patients with isolated closed limb injuries are excluded, as are patients over 65 years with isolated fracture neck of femur or pubic ramus fracture. All other isolated closed femoral injuries are included. The details of the data collection process have been described in detail elsewhere. Briefly, data are collated by trained staff in participating hospitals and submitted via the TARN Electronic Data Collection and Reporting (EDCR) system (ref ). Each submission is checked for consistency and accuracy by trained coders at the University of Manchester. All injuries are coded using the Abbreviated Injury Scale 1998 Dictionary which allocates each injury a severity code between 1 (minimal) and 6 (maximal). [11] AIS severity coding is derived from the precise injury descriptions given by imaging, operative and post mortem reports. Adult (age > 15 yrs at time of injury) patients presenting between 2001 and 2008 to TARN participating hospitals were included in the study dataset if they had a Glasgow Coma Score (GCS) less than 15 at presentation or any head injury with AIS severity code 3 and above. Only cases with known final outcome were selected.

### Variables

#### Main Exposure

The extent of intracranial bleeding was determined from the AIS code. [11] IB was coded as epidural (EDH) subdural (SDH) and intraparenchymal (IPH). Each type was coded as absent, present small (AIS4), present large (AIS5) or present size unspecified (AIS4), referred as *"no further specification" *(NFS) in this paper. There are differences in the volume of blood that attract "small/large (AIS4/5)" codes depending on the site of bleeding (Additional file 1). Subarachnoid haemorrhage (SAH) was coded as present or absent. Data about the size of the haematoma refer to the "worst" CT scan information available.

Potential confounders of the relationship between size of bleeding and patient outcome were selected for the multivariable analysis. These variables were: age, GCS, SAH, brain contusions, brain swelling, petechial haemorrhages, presence of other brain injuries (skull fractures and any brain lesion no further specified), presence of extracranial injuries (AIS with severity score >2), and whether or not the TBI patient has been treated at a neurosurgical unit (NSU). These variables have previously been reported to be associated with poor outcome. [4,12-14]

Other variables reported for descriptive purposes of the sample were: gender, cause of injury (road traffic crash, fall <2 m, fall > 2 m) and Injury Severity Score (ISS). ISS is a summary of the overall severity of anatomical injury for each patient. [15] It has an ordinal scale from 1-75 and is derived from the AIS severity scores for each injury.

The main outcome was in hospital mortality. We also explored the association between size of bleeding and evacuation of haematoma.

### Analysis

Deciding which variables should be considered confounders and which should be considered mediators that are on the causal pathway between bleeding and outcome requires a conceptual framework. We could consider as confounders all variables shown to be associated with poor prognosis in TBI, such as age, severity of the TBI (as defined by GCS) and other CT scan abnormalities. However, some of these variables (e.g. brain swelling) might be on the causal pathway between bleeding and patient outcome, and others (e.g. GCS) might be a consequence of intracranial bleeding, though not on the causal pathway. Adjusting for these variables would attenuate a true association between bleeding and outcome. Because of the uncertainty in determining which factors are confounders and which are on the causal pathway, we analysed the data from two conceptual frameworks, in the hope that the two different analyses would provide a better understanding of the association between IB and outcome. The first includes all potential confounders, the second, excludes those variables that could be on the causal pathway between IB and patient outcome. Each exposure and confounding variable was entered into a multivariable logistic regression analysis to analyse its relationship with outcome. A first analysis considered no bleeding as the baseline category and mortality and haematoma evacuation as outcomes. Because we were interested in quantifying the mortality risk associated with large, as opposed to small IB, we also conducted a second analysis evaluating the effect of IB size on mortality using small IB as the baseline. To determine the functional form of the predictors age and GCS in the model, fractional polynomials, quadratic and cubic spline and Lowess smoothing were explored.

## Results

Between 2001 and 2008 15,754 adult patients meeting study inclusion criteria for TBI presented to TARN hospitals and were submitted. In 1792 patients, the GCS on arrival at the first hospital was missing. The remaining 13,962 were used for this study.

Table 1 describe the characteristics of the study population. Almost three quarters of the patients were male. The median age was 41 years old, the median GCS was 13 and the median ISS was 18. The commonest mechanism of injury was road traffic crashes and in-hospital mortality was 22%. A total of 6445 patients (46%) had some type of IB (EPH, SDH, IPH or SAH). Of these patients 2,922 (45%) had one type of IB, 1018 (16%) had two types of IB, 1619 (25%) had three types of IB and 886 (14%) had four types IB.

**Table 1:** Demographics

	**All patients**	**EDH haemorrhage**	**SDH haemorrhage**	**IPH haemorrhage**	**SAH**	**No bleeding**
**N**	13962 (100%)	3140 (22.5%)	4204 (30.1%)	2990 (21.8%)	3025 (21.7%)	7517 (53.8%)
**NFS**		2,185 (70%)	1985 (47%)	2193 (73%)		
**Small**	NA	536 (17%)	1168 (28%)	321(11%)	NA	NA
**Large**		419 (13%	1051 (25%)	476 (16%)		
**Median Age**	40.7	43.4	48.9	47.1	46.6	37.8
**Male**	10229 (73.3%)	2352 (74.9%)	3050 (72.5%)	2187 (73.1%)	2257 (74.6%)	5456 (72.6%)
**Female**	3733 (26.7%)	788 (25.1%)	1154 (27.5%)	803 (26.9%)	768 (25.4%)	2061 (27.4%)
**Median GCS**	13	11	10	11	8	14
**Median ISS**	18	25	25	25	25	13
**RTC**	6125 (43.9%)	1053 (33.5%)	1337 (31.8%)	1025 (34.3%)	1299 (42.9%)	3756 (50.0%)
**Fall more > 2 m**	2312 (16.6%)	753 (24.0%)	1026 (24.4%)	690 (23.1%)	715 (23.6%)	892 (11.9%)
**Fall <2 m**	2706 (19.4%)	720 (22.9%)	1119 (26.6%)	763 (25.5%)	561 (18.5%)	1238 (16.5%)
**Other**	2819 (20.2%)	614 (19.6%)	722 (17.2%)	512 (17.1%)	450 (14.9%)	1631 (21.7%)
**Non NSU**	7907 (56.6%)	1523 (48.5%)	2044 (48.6%)	1634 (54.6%)	1426 (47.1%)	4813 (64.0%)
**NSU**	6055 (43.4%)	1617 (51.5%)	2160 (51.4%)	1356 (45.4%)	1599 (52.9%)	2704 (36.0%)
**Mortality**	3065 (22.0%)	869 (27.7%)	1380 (32.8%)	950 (31.8%)	1222 (40.4%)	1098 (14.6%)

NFS: No further specified; GCS: Glasgow coma scale; ISS: Injury severity score; RTC: Road traffic collision; NSU: Neurosurgical unit; EDH: Extradural haematoma; SDH: Subdural Haematoma; IPH: Intracerebral Haemorrhage

SDH was the most common type, present in 30% of the patients. EDH, IPH and SAH were present in 22% each. Among patients with IB, the size (either large or small) was reported in 30% of those with EDH, in 53% with SDH and in 27% of patients with IPH. Patients with IB were in general older, with more severe TBI (as defined by GCS) and had higher in-hospital mortality. Among the different types of IB, patients with EDH were youngest, and those with SAH had the highest in-hospital mortality. Patients with IPH were less frequently hospitalized in services with neurosurgery (NSU). Patients with missing GCS, and therefore excluded, were similar to those included in the analysis but there was a slightly larger proportion of males (81% vs. 73%) with a higher median ISS (25 vs.18).

### Relationship between age and GCS with mortality

**Figure 1:**
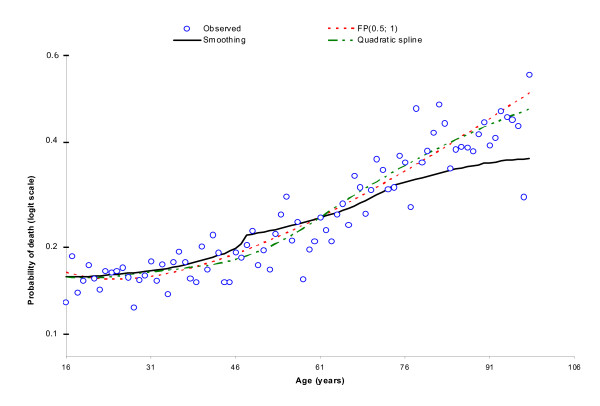
**Functional form for Age**.

**Figure 2:**
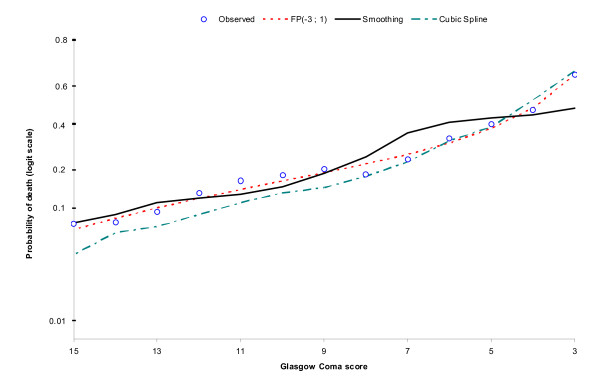
**Functional form for GCS**.

Figures 1 and 2 show the fit of the three functional forms to the observed data. It can be seen that fractional polynomials (FP) fit the data well for both Age and GCS, therefore they were included in this way in the analysis.

For Age the optimal functional form is the sum of square root age and age, for GCS it is the sum of inverse GCS cubed and GCS.

#### In-hospital Mortality

Table 2 shows the unadjusted and adjusted effect (odds ratio) for mortality of the different types, and size, of IB.

**Table 2:** Odds ratios (95% confidence intervals) for mortality according to haemorrhage size

		**Unadjusted **	**Adjusted model 1 †**	**Adjusted model 2‡**
	No EDH	1	1	1
EDH haemorrhage	NFS	1.77 (1.60 to 1.96)	1.28 (0.84 - 1.93)	1.27 (0.89 - 1.83)
	Small	0.57 (0.43 to 0.74)	0.67 (0.47 - 0.95)	0.61 (0.45 - 0.83)
	Large	1.61 (1.29 to 2.01)	1.85 (1.36 - 2.51)	2.11 (1.62 - 2.75)

	No SDH	1	1	1
SDH haemorrhage	NFS	1.75 (1.57 to 1.96)	0.98 (0.71 - 1.35)	1.05 (0.80 - 1.40)
	Small	1.31 (1.13 to 1.53)	0.99 (0.81 - 1.22)	1.21 (1.02 - 1.44)
	Large	6.30 (5.50 to 7.21)	3.36 (2.76 - 4.08)	7.09 (6.01 - 8.37)

	No IPH	1	1	1
IPH haemorrhage	NFS	1.79 (1.61 to 1.98)	1.13 (0.75 - 1.69)	1.40 (0.98 - 1.99)
	Small	0.88 (0.65 to 1.19)	0.89 (0.61 - 1.30)	0.83 (0.60 - 1.15)
	Large	4.19 (3.46 to 5.06)	3.10 (2.38 - 4.04)	3.45 (2.74 - 4.33)

Variables included in the model: † Age, GCS, NSU, EDH, SDH, IPH, Brain contusion, Swelling, Petechial, SAH, Other brain injuries, Extracranial injuries.

‡ Age, NSU, EDH, SDH, IPH, Brain contusion, Petechial, Penetrating, SAH, Other brain injuries, Extracranial injuries.

##### Unadjusted analysis

IB either coded as large or as NFS in all locations were associated with an increased risk of mortality in comparison with no bleeding. Large SDH and large IPH were associated with a worse prognosis, with an odds ratio for mortality of 6.30 (*95%CI 5.50-7.21*) and 4.19 (*95% CI 3.46-5.06*) respectively. Small SDH were the only small lesions associated with an increase in mortality.

##### Adjusted analysis

There was strong evidence of an association with mortality for all the potential confounder variables (age, GCS, presence of extracranial injury, treatment at a NSU, brain contusion, brain swelling, petechial haemorrhages, SAH and other brain injuries) so they were all included in the multivariable model.

After adjustment for confounding variables, large IB irrespective of location was associated with an increased risk of mortality. The odds ratio for large SDH was halved after adjustment (3.36 *95% CI: 2.76-4.08*), the odds ratio for large IPH was slightly attenuated (3.10 *95% CI: 2.38-4.03*) and the association between large EDH and mortality remained virtually unchanged (1.85 *95% CI: 1.36-2.51*). After excluding GCS and brain swelling from the multivariable analysis (model 2 in table 2), large IB remained the only ones with a significant association with mortality, with values that were more extreme than the odds ratio reported in the fully adjusted models.

#### Evacuation of haematoma

Table 3 shows the unadjusted and adjusted effect (odds ratio) for haematoma evacuation of the different types, and size, of IB.

**Table 3:** Odds ratios (95% confidence intervals) for haematoma evacuation according to haemorrhage size

		**Unadjusted **	**Adjusted model 1†**	**Adjusted model 2‡**
	No EDH	1	1	1
**EDH hemorrhage**	NFS	4.69 (4.02 to 5.49)	2.78 (1.75 - 4.44)	2.95 (1.85 - 4.70)
	Small	3.96 (2.98 to 5.20)	2.99 (2.15 - 4.20)	3.08 (2.22 - 4.27)
	Large	22.56 (18.05 to 28.16)	25.58 (18.80 - 34.81)	28.87 (21.27 - 39.20)

	No SDH	1	1	1
**SDH hemorrhage**	NFS	6.43 (5.40 to 7.65)	5.58 (3.78 - 8.25)	5.77 (3.91 - 8.50)
	Small	3.96 (3.14 to 4.98)	3.29 (2.50 - 4.33)	3.59 (2.73 - 4.72)
	Large	13.70 (11.40 to 16.47)	15.47 (11.88 - 20.13)	19.40 (15.07 - 24.97)

	No IPH	1	1	1
**IPH hemorrhage**	NFS	3.54 (3.07 to 4.08)	0.58 0.36 - 0.95)	0.58 (0.36 - 0.95)
	Small	0.82 (0.44 to 1.40)	0.617 (0.31 - 1.22)	0.66 (0.34 - 1.29)
	Large	1.91 (1.36 to 2.63)	0.91 (0.57 - 1.44)	1.04 (0.66 - 1.63)

Variables included in the model: † Age, GCS, NSU, EDH, SDH, IPH, Brain contusion, Swelling, Petechial, SAH, Other brain injuries, Extracranial injuries.

‡ Age, NSU, EDH, SDH, IPH, Brain contusion, Petechial, Penetrating, SAH, Other brain injuries, Extracranial injuries.

##### Unadjusted analysis

IB from all the locations and from all the categories (large, small and NFS) were associated with an increased risk of evacuation, except for small IPH. EDH and SDH showed the largest odds ratio (22.6 and 13.7 respectively)

##### Adjusted analysis

After adjusting for all potential confounding variables, there was an increased risk of haematoma evacuation for both SDH and EDH. The magnitude of the association was larger for large haematomas, intermediate for those coded as NFS and smallest for the small ones. The odds ratio for large EDH and SDH were, respectively, 25.58 (*95% CI: 18.80-34.81*) and 15.47 (*95% CI: 11.88-20.13*). After multivariate analysis none of the categories of IPH remained positively associated with evacuation. Similar results were obtained when excluding GCS and brain swelling from the multivariable adjustment.

#### Comparison between large and small haemorrhages

In table 4 it can be seen that large IB, wherever the location, were associated with an increased risk of mortality, in comparison with small IB lesions. After adjusting for potential confounders (model 1) the odds ratio for mortality was 2.86 (*95% CI: 1.86-4.38*) for large EDH, 3.41 (*95% CI: 2.68-4.33*) for large SDH and 3.47 (*95% CI: 2.26-5.33*) for large IPH. Patients with EDH coded as NFS had an odds ratio for mortality of 1.89 (95% CI: 1.20-2.99) in comparison with those with small EDH. There was no strong evidence of increased risk of mortality for those patients with SDH or IPH coded as NFS when compared with patients with the corresponding lesions coded as small.

**Table 4:** Odds ratios (95% confidence intervals) for mortality with small haemorrhages as baseline

		**Adjusted odds ratio †**
	Small	1
EDH haemorrhage	No EDH	1.49 (1.05 - 2.12)
	NFS	1.89 (1.20 - 2.99)
	Large	2.86 (1.86 - 4.38)

	Small	1
SDH haemorrhage	No SDH	1.07 (0.85 - 1.35)
	NFS	0.99 (0.72 - 1.37)
	Large	3.41 (2.68 - 4.33)

	Small	1
IPH haemorrhage	No IPH	1.23 (0.84 - 1.80)
	NFS	1.39 (0.84 - 2.28)
	Large	3.47 (2.26 - 5.33)

Variables included in the model: † Age, GCS, NSU, EDH, SDH, IPH, Brain contusion, Swelling, Petechial,, SAH, Other brain, Extracranial injuries.

## Discussion

This analysis of over 13,000 patients with TBI showed that patients with a large EDH, SDH or IPH have a substantially higher mortality than patients with either no bleeding or a small bleed. Even after adjusting for other CT findings, such as contusions and brain swelling, and other potential confounding variables, such as age and GCS, large bleeds substantially increased the probability of death. Patients with large IPH or large SDH had more than a threefold increased in mortality odds in comparison with patients with small IB in the same location, while large EDH showed more than a doubling in the mortality odds in comparison with patients with small EDH. Small IB were not associated with an increased in mortality after adjustment for other potentially confounding variables. Patients with IB coded as NFS had generally a risk which was intermediate between that reported for patients with large and the one reported for patients with small IB.

The frequency of IB after a TBI varies according to the inclusion criteria of the different studies. The incidence of IB in our analysis was higher than other series because of the TARN inclusion criteria. TBI patients are included if they die at any point during admission, stay in hospital for longer than 3 days, require intensive or high dependency care or require inter - hospital transfer for specialist care. Studies that also included hospitalized TBI patients reported similar frequencies of IB as the one we reported. For example in the CRASH Trial which was the largest trial conducted among TBI patients, 56% of the patients have some type of IB and 27% presented a subarachnoid haemorrhage which is similar to the 22% incidence reported in this study. [4] In the IMPACT study, which included 9 randomised clinical trials in TBI patients, the range of frequency for EPH and SDH was 7-20% and 20-36% respectively, which is similar to the frequency reported in our study. [16] Our results are consistent, but more precise, than those of previous studies showing that IB is associated with increased mortality. There has not been any systematic review describing the association between size of IB and prognosis in TBI but a comprehensive review has been reported in the Guideline for the Surgical Management of Traumatic Brain Injury". [17] In this guideline bleeding size is taken into account to recommend surgical evacuation. However, the evidence presented in the guideline is very limited. For EDH the guideline reported only seven studies that evaluated the effect of size on outcome. The median of patients included was 74 (range: 22-200). In relation to SDH only seven studies reported on the effect of size with a median number of patients of 91 (range 23-206). For IPH seven studies were reported, with a median of 85 patients included (range 23-321). Furthermore in only a few studies mortality or disability were considered as outcomes and, many of the studies included selected samples (e.g. only surgical patients) and were retrospective analysis of one centre database.

Our findings are also in keeping with the results of previous publications showing that SDH is associated with a much larger increased in the probability of death than EDH. [17]

We found that only large IB, wherever the location (EDH, SDH or IPH) are associated with worse outcome and that large IB are associated with an increased risk of death in comparison with small IB. Further studies should also evaluate the effect of size on disability using outcomes such as the Glasgow Outcome Scale.

The strength of our analysis is that it included more than 13,000 patients with traumatic brain injury and so the precision of our estimates of the risk associated with IB is high. We also adjusted for most of the relevant potential confounding variables.

An important limitation of this study is that in a large proportion of patients it was not reported whether the IB was small or large. These patients, with size coded as NFS, presented intermediate risk between patients with small and large lesions. Another limitation is that we did not have information on pupil reactivity which has been shown to be an important prognostic factor. A further limitation is that we had no continuous measurements of the size of the bleeding, nor did we analyse on the timing of the CT scan. Although 90% of patients were included in the analysis, some were excluded because of a lack of data on GCS. Nevertheless, the characteristics of the excluded patients were similar to those included, making bias unlikely. Finally, size of bleeding is only one of the prognostic factors in patients with TBI and IB. In this study we were able to consider some of other prognostic factors such as TBI severity (GCS) and brain swelling, but other such as the localization of the bleeding were not available and were not included in this analysis. It is plausible that the effect of IB will vary according to the localization, as the functional outcome would be influenced by the brain region affected,. Future studies should explore the different effect on functional outcomes according to the localization of the IB.

There is some evidence that bleeds could enlarge in the 24-48 hours after injury. Oertel and colleagues studied patients in whom two CT scans were obtained within 24 hours of injury to determine the prevalence of progressive bleeding. [18] Among patients who had their first scan within 2 hours of injury, 49% had radiological evidence of progressive bleeding. Yadav and colleagues scanned TBI patients at hospital admission and 24 hours later, and found that 16% of 262 parenchymal haematomas and contusions increased in size in the first 24 hours. [19] Similarly, Sullivan et al found that traumatic epidural haemorrhages enlarged in 23% of 160 TBI patients treated non-operatively. [20] More recently Narayan and colleagues reported a study in which they included patients with TBI and IPH confirmed by CT scan of = 2 ml. They repeated the CT scan at 24 and 72 hours and found that in 51% of the included patients IPHs expand in the first 24 hours. Although these studies provide estimates of the occurrence of intracranial bleeding and expansion they all have limitations. All included patients who have an abnormal initial CT scan and there is little information on the proportion of patients that develop new intracranial bleeds in the first 24 hours who have the potential to benefit from early treatment. Further studies are needed to clarify the timing of IB expansion. A large cohort of TBI patients with an early CT scan and a second CT scan within 24-48 hours including patients with a range of TBI severity is needed to clarify the natural history and prognostic role of traumatic progressive IB.

## Conclusion

In conclusion in this analysis we found an association between size of intracranial bleeding and mortality in TBI patients. However, the limitation of our data, such as the large proportion of missing data and lack of data on other confounding factors such as localization of the bleeding make the results of this report only explanatory. Furthermore we were only able to evaluate the effect of IB in mortality. Future studies with more complete data should confirm or refute our results and also evaluate the effect of IB size on functional outcomes

Finally the effect on mortality of interventions that reduce the extent of intracranial bleeding would only be able to establish through prospective randomised controlled trials.

## Competing interests

The authors declare that they have no competing interests.

## Authors' contributions

PP IR and FL designed the study. OB conducted the analysis. All the authors contributed to the final version of the manuscript.

## Pre-publication history

The pre-publication history for this paper can be accessed here:



## Supplementary Material

Additional file 1 **Appendix 1 AIS 1990 revision, update 1998**. This appendix describes the Abbreviated Injury Scale (AIS). Click here for file
