# Supplementary material for: Intracranial bleeding in patients with traumatic brain injury: A prognostic study
Source: BMC Emerg Med. 2009 Aug 3;9:15. doi: 10.1186/1471-227X-9-15 (PMC2735732; doi:10.1186/1471-227X-9-15)
Supplement: Additional file 1 — Appendix 1 AIS 1990 revision, update 1998. This appendix describes the Abbreviated Injury Scale (AIS). [file 1471-227X-9-15-S1.doc]

**Appendix 1**

**AIS 1990 revision, update 1998**

**Small EDH**

Cerebellum ≤ 30cc

Cerebrum ≤ 50mls

**Small SDH**

Cerebellum ≤ 30cc

Cerebrum ≤ 50cc

**Small IPH**

Cerebellum (≤15cc; or 3cm diameter)

Cerebral ≤ 30cc; ≤ 4cm diameter

**Large EDH**

Cerebellum >30cc, > 2cm diameter/ thick, massive / extensive, bilateral

Bilateral cerebral

Cerebral > 50 cc, >1cm thick, massive / extensive

**Large SDH**

Cerebellar > 30cc, > 2cm diameter/ thick, massive /extensive, bilateral

Bilateral cerebral

Cerebral > 50 cc, >1cm thick, massive / extensive

**Large IPH**

Cerebellar > 15cc; >3cm diameter, bilateral

Bilateral cerebral

Cerebral > 30cc / 4cm diameter

**EDH NFS**

Epidural or extradural haematoma (haemorrhage) to cerebellum not further specified

Cerebrum epidural or extradural haematoma/haemorrhage not further specified,

**SDH NFS**

Cerebellar subdural haematoma not further specified

Cerebrum sub dural haematoma not further specified

**ICH NFS**

Intracerebellar including petechial and subcortical haematoma (haemorrhage) not further specified

Cerebrum intracerebral haematoma not further specified
